# Bump Feeding Improves Sow Reproductive Performance, Milk Yield, Piglet Birth Weight, and Farrowing Behavior

**DOI:** 10.3390/ani13193148

**Published:** 2023-10-09

**Authors:** Keiven Mark B. Ampode, Hong-Seok Mun, Eddiemar B. Lagua, Veasna Chem, Hae-Rang Park, Young-Hwa Kim, Chul-Ju Yang

**Affiliations:** 1 Animal Nutrition and Feed Science Laboratory, Department of Animal Science and Technology, Sunchon National University, Suncheon 57922, Republic of Korea; 2 Department of Animal Science, College of Agriculture, Sultan Kudarat State University, Tacurong 9800, Philippines; 3 Department of Multimedia Engineering, Sunchon National University, Suncheon 57922, Republic of Korea; 4 Interdisciplinary Program in IT-Bio Convergence System (BK21 Plus), Sunchon National University, Suncheon 57922, Republic of Korea; 5 Interdisciplinary Program in IT-Bio Convergence System (BK21 Plus), Chonnam National University, Gwangju 61186, Republic of Korea

**Keywords:** late gestation, backfat thickness, postural changes, birth weight, litter size

## Abstract

**Simple Summary:**

Genetic advancements have increased litter size, resulting in lower birth weights due to intrauterine nutrient competition. Low-birthweight piglets have higher mortality rates and face growth and reproductive challenges. Birth weight and litter size uniformity are crucial factors in commercial swine production, as they affect postnatal growth and piglet survival. Prolonged farrowing duration, associated with large litter size, contributes to stillborn piglets. A recent study developed a feeding technology in the late gestation period in sows to improve reproductive performance, farrowing behavior, and piglets’ growth performance during the lactation period. The findings of this study provide valuable information for optimizing sow nutrition and improving swine production efficiency.

**Abstract:**

The late gestation period is crucial for fetal growth and development, impacting swine enterprises’ profitability. Various nutritional strategies have been explored to enhance reproductive performance in sows, but findings regarding birth weight and litter size have been inconsistent. This study investigated the effects of increased feeding allowance during the late gestation period on the reproductive performance and farrowing behavior of primiparous and multiparous sows. A total of 28 sows (Landrace × Yorkshire) were used in this experiment, and fed 2.50 kg/d or 3.50 kg/d from 84 days of gestation until farrowing. Farrowing behavior was monitored using a DeepEyes^TM^ M3SEN camera. The data were analyzed using the 2 × 2 factorial within Statistical Analysis System (SAS, 2011, Version 9.3) software. The results indicated that regardless of the parity number, sows fed a high diet exhibited a numerical increase in the total number of born piglets and a significant increase in milk yield (*p* = 0.014) and piglet birthweight (*p* = 0.023). Backfat thickness loss was significantly higher in sows with a 2.50 kg feeding allowance (*p* = 0.022), and the total number of piglets born, live births, and litter size were numerically higher in sows fed 3.50 kg per day. Moreover, stillborn piglets, mortality rate, and re-estrus days were numerically lower in sows with a high feeding allowance. The diet and parity did not individually affect the average duration of farrowing and farrowing intervals. However, the duration of postural changes in sows after farrowing was significantly reduced (*p* = 0.012). The principal component analysis revealed 81.40% and 80.70% differences upon partial least-squares discriminant analysis. Therefore, increasing feeding allowance during the late gestation period, regardless of parity, could positively influence sows’ reproductive performance and piglets’ growth performance during the lactation phase.

## 1. Introduction

Genetic selection, health management, and nutrition improvements have resulted in unprecedented sow productivity. Over the past few years, intensive genetic improvement has increased litter sizes [1]. However, this increase in litter size has led to a decrease in birth weight within each litter due to greater competition for nutrients in the intrauterine environment [1,2,3]. Low-birthweight piglets have a higher mortality risk during the nursery period, particularly when their colostrum intake is reduced [4]. These piglets also experience reduced weaning weight, resulting in lower market weight and decreased reproductive performance [5,6,7,8]. The birth weight and uniformity of litter size at birth are crucial production variables in commercial swine production [9], as they strongly correlate with postnatal growth rate, piglet survival during lactation, and muscle development at the time of harvest [10,11,12].

On the other hand, the duration of farrowing is positively correlated with a large litter size [13], resulting in an increase in the number of stillborn piglets primarily due to perinatal asphyxia [14]. Researchers and practitioners have continuously studied and implemented mechanisms to mitigate these effects, enhancing the nutritional intake and the productive and reproductive indices of gilts or sows in intensive production systems [15]. Nutrient intake during gestation greatly influences reproductive performance and the release of reproductive hormones in sows [16]. The key indicators for assessing productive potential in livestock production include litter size, birth weight, growth, and mortality rates, all of which are associated with parity order [17].

In recent decades, various nutritional strategies have been explored to improve piglet birth weight and optimize reproductive performance in sows. These strategies include L-carnitine supplementation in early gestation [18] and the manipulation of energy levels or lysine during the late gestation phase [19]. Moreover, Moreira et al. [20] reported that L-arginine supplementation during the final third of the gestation period improves litter uniformity and the physical characteristics of neonatal piglet thermoregulation. Another strategy, called ‘bump feeding’, involves increasing the sow’s daily feed intake by about 1 kg from day 90 of gestation to farrowing [21]. The goal is to provide the gestating sow with the extra energy and amino acids needed in late gestation to support the conceptus’s exponential growth [22]. Increasing the average daily feed intake (ADFI) in the later stages of pregnancy contributes to mammary gland development, milk production [23,24], fetal growth [25,26], and the development of piglet thermoregulation.

The advantages of implementing the bump feeding practice during the late gestation period remain unclear. Shelton et al. [27] reported that gilts with high feed intake during late gestation had increased piglet birthweight. However, Soto et al. [28] reported that bump feeding did not significantly affect piglet birth weight in sows. Therefore, it is important to explore nutritional strategies that support maternal and fetal development while minimizing the risk of compromising reproductive performance. This study investigated the impact of increasing sows’ feeding allowance during late gestation, and determined its effect on their physiological condition, reproductive performance, milk yield, and farrowing behavior.

## 2. Materials and Methods

### 2.1. Animals and Diets

The study was conducted from November 2022 to March 2023 at Sunchon National University’s experimental swine facility. A total of 28 sows (Landrace × Yorkshire) were used in the experiment and housed in individual gestation crates with an area of 2.02 × 0.70 m with autoloading feeding systems and *ad libitum* access to water. Each treatment group comprised 14 experimental animals, with the sows being equitably allocated. Specifically, within each group, there were seven sows in their first parity and seven multiparous sows, exhibiting parity numbers ranging from 2 to 8. On average, the multiparous sows in each group had a parity number of 3.69.

Experimental animals were submitted to flush feeding about 20 days before artificial insemination (AI). Moreover, synthetic progesterone was administered orally daily for estrus synchronization. Following a 24 h interval, sows were injected with PMSG (pregnant mare’s serum gonadotropin) to stimulate livestock ovulation before artificial insemination. After 72 h of PMSG administration, all sows were injected with hCG (human chorionic gonadotropin) to promote progesterone production, facilitating the preparation of the uterine lining for implantation. Standard farm management protocols were adhered to for the sows and piglets. The experimental animals were housed in a controlled environment with an ambient temperature of 22 °C and relative humidity of 64%. A more detailed description of synchronizing estrus, the procedure for performing AI, and the sensors used in collecting housing conditions have been discussed in previous studies [29,30].

Pregnancy diagnosis was performed on all sows at 21 and 35 days post-mating using a pig ultrasound diagnosis scanner (Easy Scan Gold, Dongjin BLS Co., Ltd., Gwangju-si, Republic of Korea). At day 84 of gestation, 28 sows (Landrace × Yorkshire) were selected and assigned to two dietary treatments (Table 1). The gestation diet was based on corn-soybean meal, with 3200 digestible energy (Kcal/kg), 16% crude protein (CP), and 0.50% standardized ileal digestible lysine (SID Lys). The composition of the diet can be accessed in a previous study [30]. The control group was fed 2.50 kg a day until farrowing, while the treatment group’s feed allowance was increased to 3.50 kg daily from 84 days of pregnancy to farrowing. All sows were individually fed five times a day at 8:00 am, 11:00 am, 2:00 pm, 5:00 pm, and 10:00 pm with their corresponding feeding amount.

### 2.2. Reproductive Performance

On day 107 of gestation, all sows were individually weighed, moved into the farrowing house, and kept in an individual farrowing crate measuring 2.02 × 0.70 m with an autoloading feeding system. After farrowing, all sows were fed with a commercial diet and received the same amount of feed during the lactation period until day 28, and their feed intake was recorded (Table 2). 

The birth weight of the piglets, the total number of piglets born, piglets born alive, and those stillborn were recorded for each litter within 24 h after birth. The mummified fetuses and stillbirths were not weighed; however, the number was recorded to be included in the total number of piglets born [1]. Obstetric assistance and oxytocin hormone were not provided to the sows during farrowing. The piglets were individually weighed again at 3 days old to determine the sow’s milk yield by calculating the piglets’ litters body weight gain multiplied by 4.2 [31,32]. After weighing, piglets were cross-fostered according to their dietary treatment to standardize a litter size of 11–13 piglets per sow. No piglet mortality was recorded within 3 days after farrowing. After 3 days from birth, the piglets had *ad libitum* access to creep feeds and clean drinking water. The pre-weaning mortality (%) was calculated by dividing the total number of piglets in each treatment by the number of piglets that died after cross-fostering [33]. The sows were weighed individually after 28 days of lactation phase. 

The number of re-estrus days was determined, beginning 3 days after weaning. The estrus detection was conducted twice daily (09:00 and 15:00 h) using the two methods. The first method is the fence line contact with a boar, and sows were determined to be in estrus when they displayed the standing reflex in response to the back pressure test [33,34] and riding on the back test. The second method involved a digital infrared thermal imaging camera FLIR E76 with the emissivity set at 0.95, and body temperature from the anus and vulva was taken at a distance of 1 m from the animals. Sows were considered estrus when their average body temperature was 35 °C above [30]. 

### 2.3. Body Condition Score and Backfat Thickness Determination

Backfat thickness (mm) was measured ultrasonically and using a sow backfat caliper. A digital backfat measuring device (Minitube Backfat meter, Dongjin BLS Co., Ltd.) and sow backfat caliper were used to assess backfat thickness at the level of the last rib on each side of the sow at the P_2_ point, which is 65 mm from the midline [35,36], and the mean values were used for analysis. The body condition score (BCS) was determined through visual estimation and by pressing the hipbone and backbone of the sow, using a scale of 1 to 5, where 1 indicates visually thin and 5 indicates that the hipbone and backbone cannot be felt. The backfat thickness and BCS loss were determined by calculating the difference between backfat thickness at 107 days of gestation and backfat thickness and BCS at weaning. 

### 2.4. Farrowing Behavior and Postural Changes

Farrowing can be a stressful and painful event for sows, and the postural changes and duration of farrowing are indicators of stress and dystocia. Each farrowing crate was equipped with a DeepEyes™ camera (M3SEN, Seoul, Republic of Korea) pointing downward to capture a top view of the farrowing area at 2.23 m off the ground. The area was video recorded for 24 h. DeepEyes™ M3SEN cameras are the first artificial intelligence sow management system that utilizes advanced technology to detect and immediately notify farmers of sow labor, dystocia, health status, and postural changes in real time. The frequency and duration of standing before farrowing were collected from the average data per day from 4 days before farrowing until 2 days before farrowing. On the other hand, the frequency and duration of standing after farrowing were collected from the average data per day from 2 days after farrowing until 4 days after farrowing.

### 2.5. Statistical Analysis

The data were analyzed using the 2 × 2 factorial (diet level and parity) of the Statistical Analysis System (SAS, 2011, Version 9.3, SAS Institute, Cary, NC, USA) software. The difference in the sows’ reproductive performance was analyzed using a principal component analysis and a partial least-squares discriminant analysis with MetaboAnalyst 5.0 and Tbtools software (v2.008) [37]. All data in the investigation were analyzed with a confidence limit set at 95% (*p* < 0.05).

## 3. Results

### 3.1. Physiological Condition

Backfat thickness (mm) and body condition score (BCS) during the 84 and 107-day gestation period had no significant effect on the diet and parity. Although no significant difference was observed, sows fed with 3.5 kg per day during the late gestation period exhibited a numerical increase in backfat thickness. Furthermore, the interaction between diet and parity significantly affected the sows’ physiological condition (Table 3). High diet during the late gestation period significantly affected sow backfat thickness and BCS, while sows fed a low diet had a higher backfat loss at weaning.

### 3.2. Sows’ Reproductive Performance

Diet had no significant effect on the reproductive performance of sows in most parameters, except for feed intake. Although no significant difference was observed, sows receiving higher amounts of feed during late gestation exhibited less body weight loss during the weaning period than those on a lower diet. Additionally, sows fed with 3.50 kg per day displayed numerically higher values for the total number of piglets born, live birth, livability at farrowing and weaning, and litter size weaned. The data also indicated that sows with a feeding allowance of 3.50 kg per day had lower values for the total number of mummified piglets, stillbirth, and mortality, and re-estrus days (Table 4).

**Figure 1:**
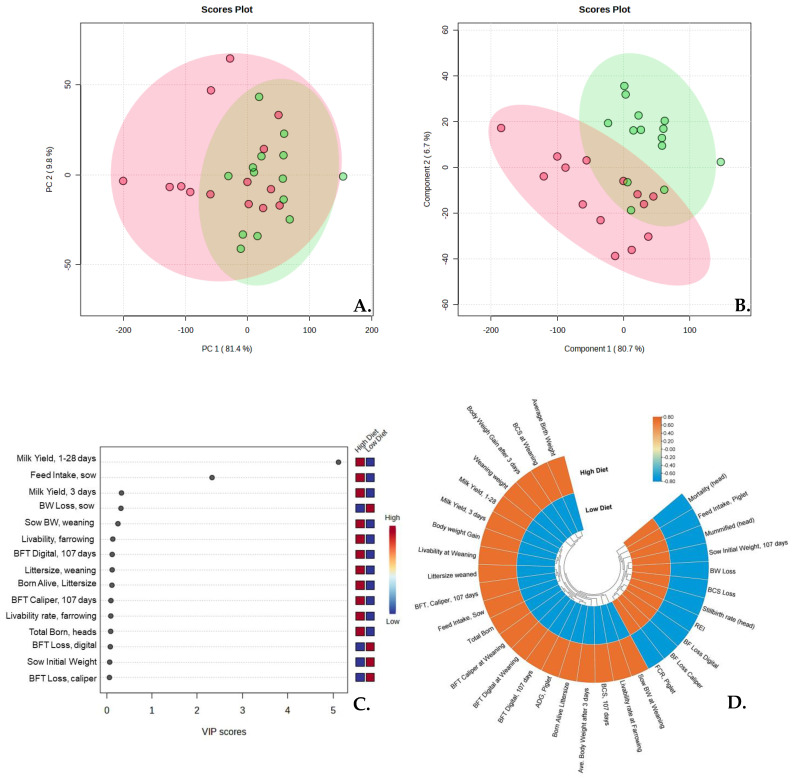
The effects of bump feeding on the sows’ physiological condition, reproductive performance, and piglets’ growth performance; (**A**) principal component analysis; (**B**,**C**) partial least squares discriminant analysis (PLS-DA); and (**D**) circular heatmap correlations.

Considering the *p*-value of 0.062 for litter size weaned and 0.050 for the number of re-estrus days, the diet showed the potential to increase the production of piglets per sow per year (PSY). Statistically, the parity number of sows significantly affected the initial and final weight, while no significant difference was observed in the interaction of parity and diet. Sows fed with a high diet tended to have shorter weaning-to-estrus intervals (*p* = 0.050). The principal component analysis revealed differences of 81.40% and 80.70% in the partial least-squares discriminant analysis, with a major difference in the feed intake and milk yield. Diet showed a positive correlation in some variables of reproductive performance (Figure 1). 

### 3.3. Farrowing Behavior and Postural Changes

The diet, parity, and interaction did not significantly affect sows’ average farrowing time and interval (Table 5). However, the diet significantly affects the minimum and distribution of farrowing intervals of less than 10 min for sows fed with 3.50 kg per day during the late gestation period. The diet and sows’ parity number had no significant effect on the frequency of standing before and after farrowing. Although there was no significant difference, the duration of standing in sows with high feeding allowance before and after farrowing is numerically lower compared to the sows fed with 2.50 kg per day (Table 6). 

**Figure 2:**
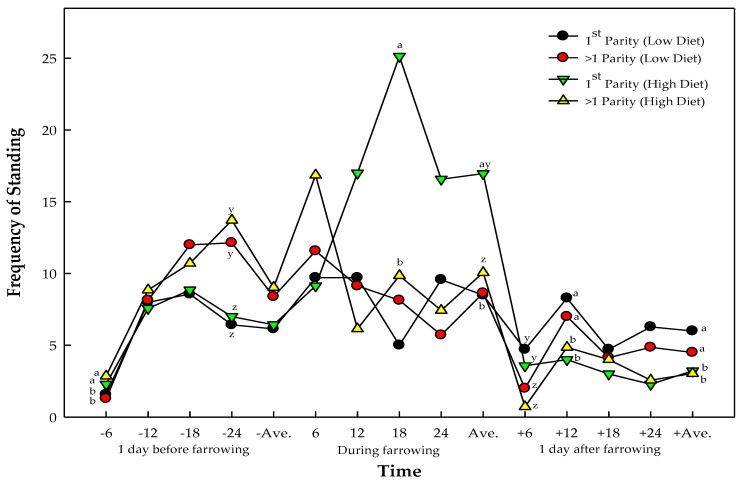
Frequency of standing of sows at different diet levels and parities, and their interactions. ^ab, yz^ letters with different superscripts within a time indicate significant difference; ^ab^ represent the effects of diet; ^yz^ represent the effects of the sows parity number.

The interaction between diet and parity significantly impacted the frequency of standing before and at the onset of farrowing (Table 7). The average frequency of standing in primiparous sows one day before and at the onset of farrowing is significantly higher in sows fed with a high diet (Figure 2). The sows with a 3.50 kg per day feed allowance during the late gestation period also exhibited a significantly lower frequency of standing than those fed with 2.50 kg per day a day after farrowing. 

### 3.4. Piglet Growth from Birth to Weaning

The piglets’ birth weight and body weight gain after 3 days were greater in sows fed with 3.50 kg than 2.50 kg per day (*p* = 0.016). The growth performance of piglets showed a positive correlation with increased feed allowance during the late gestation period. Numerically, piglets had higher body weights at weaning when sows were fed 3.50 kg per day (Table 8). Additionally, piglets from multiparous sows were significantly heavier than those from primiparous sows during the weaning period. The interaction between diet and the parity number of sows did not significantly affect the growth performance parameters. 

## 4. Discussion

In the swine industry, assessing sows’ body weight and backfat thickness throughout different stages of the production cycle is common practice. This evaluation allows for the adjustment of feeding levels to maintain the optimal body condition of sows, resulting in improved reproductive efficiency, litter performance, sow longevity, and greater mammary gland development [38,39].

In the present study, the physiological conditions of the sows during the gestation period were within the optimal range. The diet significantly affects backfat thickness, and higher backfat thickness loss was recorded in sows fed with 2.50 kg/day, while diet × parity interaction had no significant effects on the backfat thickness during the weaning period. The results of the present study were supported by Mallman et al. [1], who reported that sows with a 3.30 kg feeding allowance during the late gestation period had increased body weight, BCS, and backfat thickness. The interaction of diet and parity number in sows at 107 days of gestation period significantly influenced the BCS. Still, this did not affect backfat thickness, which was measured using a sow backfat caliper and minitube backfat measuring device. The differences in the results may be attributed to the subjective evaluation of BCS, which relied on visual estimation and pressing the sows’ hipbones and backbones. It is important to highlight that measuring backfat thickness provides a more objective and accurate means of assessing the body condition of sows [40,41] using a sow backfat caliper or digital device. 

Sows that are too fat have farrowing problems, crush piglets, have a poor appetite during subsequent lactation, and are less prolific at the next parity [42,43]. Cerisuelo et al. [44] reported that higher body reserves at farrowing may play a protective role in sow performance against the adverse effects of excessive body weight loss during lactation. Excessive loss in body weight and backfat thickness prolonged the number of days of weaning–estrus intervals, increased the incidence of anestrus, and reduced farrowing rate [45,46,47]. Thus, achieving the ideal sow body weight and condition during gestation and lactation is crucial for maximizing productivity and ensuring efficient feed utilization [41]. 

The diet had no significant effects on the initial and final body weight. However, the parity of sows significantly affected the body weight, but had no interaction effects. Multiparous sows were heavier than primiparous sows, which could be attributed to the fact that primiparous sows are still growing, while multiparous sows have already reached their optimum body weight. Although significant differences were observed in the initial weight based on the parity number in sows, these differences could not affect the experiment results because this study only investigated the reproductive performance of sows and not their growth performance. Achieving the proper body weight during gestation helps regulate weight loss during lactation and establishes optimal conditions for enhanced milk production and litter performance [48].

Piglets with a low birth weight in large litters are at a greater risk of not obtaining at least 200–250 g of colostrum [49,50,51]. This amount is crucial for achieving adequate levels of immunoglobulins and promoting optimal growth [52], providing the energy necessary for thermoregulation, and stimulating intestinal growth and maturation [49]. Consequently, inadequate colostrum intake by newborn piglets is considered the major underlying cause of piglet deaths in the first 24 h after birth [49,53]. Incorporating a strategy that promotes the mammary glands’ growth during both gestation and lactation is essential to enhance milk production [54]. This promotion is crucial because milk synthesis occurs within the mammary epithelial cells, and the quantity of these cells directly influences the overall milk production [54]. As a result, the present study’s significant increase in milk yield significantly improved the body weight of piglets at day 3, as well as the feed conversion ratio.

The increase in sow milk yield with an average of 12.95 kg/day in the present study could be related to the nutritional requirements, energy reserves, protein synthesis, and physiological changes during the late gestation and lactation phases. The data revealed that milk yield in the present study is higher than the volumes reported by Lewis et al. [55] Cerisuelo et al. [44], and Strathe et al. [56], who reported average milk yields of 6.53, 8.12, and 11.50 kg/day, respectively. However, the data in the present study are comparable to the milk yield derived by Hawe et al. [32], who reported 13.63 kg/day. Feeding a high diet during late gestation helps the sow build up energy reserves, which can be utilized for milk synthesis during lactation.

The sows’ reproductive performance includes fertility and prolificacy, such as weaning to the first mating interval and the total number of piglets born alive [47]. In the present study, the number of piglets born alive is numerically higher in primiparous sows than in multiparous sows. Considering the initial body weight of the sows, multiparous sows are significantly heavier than primiparous sows, and receive the same amount of feed. Hence, the increase in the number of piglets born might be due to the nutritional status provided to the primiparous sows during the late gestation period. It should be noted that proper nutrition is essential for fetal development and litter size. These findings align with those reported by Carrion-Lopez et al. [57], who observed a higher number of piglets born in primiparous sows fed a high diet during the early gestation period. 

The number of stillbirth piglets in sows with high feeding allowance is numerically lower compared to those with low feeding allowance, contrary to the report of Goncalves et al. [19] and Mallman et al. [1]. A significant increase in piglets’ birthweight could be attributed to the optimum nutrient supply from sows to support the growth of fetuses. In a review, Kim et al. [54] reported that insufficient nutrient supply from sows during late gestation decreases litter uniformity. This variability in fetal weight may be mitigated by dietary supplementation such as arginine to enhance blood flow, which facilitates the production of nitric oxide in the endothelial cells lining the blood vessels, inducing vasodilation [58,59,60]. However, it is noteworthy that limited nutrient supply to the fetus is also influenced by restricted blood flow through the placenta. 

Several studies have reported that augmenting the feed allowance provided to gilts and sows from the early-to-mid gestation period improved the piglets’ birthweight compared to a restricted or low diet [1,16,61,62]. In a study cited by King et al. [61], Smits et al. [63] found that implementing a high–low–high phase feeding strategy throughout the three trimesters of primiparous sow gestation, with a total increase of 63 kg of feed, resulted in a significant increase in the average birth weight from 1.30 to 1.38 kg. Additionally, Whittemore [64] estimated that a slight 0.2 kg increase in birth weight leads to a daily gain of 24 g from birth to slaughter, corresponding to an increase in subsequent carcass weight of approximately 3 kg. 

Further, increased feed allowance during gestation results in an elevated ratio of secondary to primary muscle fibers in their offspring. This outcome brings advantages such as an enhanced growth rate and improved feed efficiency during the later stages of pig growth [25,65]. Increased maternal feed intake in the early-to-mid-gestation period may also help alleviate the negative impact of uterine crowding in highly prolific sows [65]. Nevertheless, it is important to acknowledge that these findings were derived from sows with an augmented feeding allowance during the early-to-mid gestation period. Still, these may also apply to sows with a high feeding allowance during the late gestation period. 

The preceding discussion demonstrates that as pregnancy progresses, the nutritional requirements of sows undergo significant changes [43]. Therefore, it is vital to implement feeding strategies that address sows’ specific dietary needs during gestation. To address these variations, many producers adjust their feeding regimens. During the initial 30 days of pregnancy, known as the early gestation stage, the focus is on restoring the body condition score (BCS) while influencing embryo survival and implantation [22,66,67,68,69]. This period establishes a critical foundation for subsequent development. In the mid-gestation stage, which spans days 30 to 75, the focus shifts towards facilitating body growth in young sows and replenishing the body reserves depleted during lactation in older sows [22,66,67,68,69]. This phase assumes great importance in supporting the physical development of the sow. Lastly, in the late gestation stage, approximately the final 45 days preceding farrowing, the primary emphasis lies on the growth of both the developing fetus and the mammary glands [22,66,67,68,69]. Adequate nutrition during this period is crucial for ensuring optimal fetal development, preparing for successful lactation, and improving birth weight and the uniformity of piglet size at birth [70]. 

The diet, parity, and interaction did not significantly impact the sows’ average farrowing durations and intervals. These findings are closely in line with the results obtained in previous research on various European domestic pig breeds, indicating that the average farrowing duration is typically more than 2 h per 12 piglets [71]. However, the recent results are lower than Yun et al. [72]’s findings, which reported that the farrowing duration in sows (Danish Yorkshire × Danish Landrace) raised in closed and open farrowing crates was 338.00 and 399.50 min, respectively. The average duration of farrowing time appears to be numerically greater for sows fed a high diet than those fed a low diet. This difference may be attributed to the higher total number of piglets born in the high-diet group. Oliverio et al. [13,73] have reported a positive correlation between litter size and the duration of farrowing, providing support for this observation. Nevertheless, the absence of significant differences in the duration of farrowing and the total number of piglets born implies that the reproductive performance of the sow remained uncompromised. Sows fed a high diet had more piglets delivered in less than 10 min, with no reported incidences of dystocia and uterine prolapse.

The diet and parity number in sows had no significant impact on the frequency of standing before and after farrowing. Although there is no significant effect, the duration of standing after farrowing is significantly lower in sows with a high diet. The significant increase in the frequency of standing in sows fed with a high diet at the onset of farrowing could indicate maternal responsiveness, but this can also be interpreted as an indication of discomfort, specifically in primiparous sows. Whatson and Bertram [74] reported that after the birth of piglets, the sow would often stand up and turn to inspect the newborns, engaging in sniffing and nose-to-nose contact with them. However, these observations were obtained from sows in open crates or those raised in unrestrained farrowing houses. Some studies have reported that postural changes, restlessness, and locomotion activity are signs of discomfort [72,75]. 

One possible reason for the lower postural changes in sows after farrowing is the reduction in discomfort or pain associated with completing the birthing process. After farrowing, sows may experience relief from the physical exertion and discomfort of labor, resulting in decreased postural changes. Additionally, the bonding and maternal behaviors exhibited by sows towards their newborn piglets may also contribute to reduced postural changes, as the sow focuses on nursing and caring for her piglets rather than engaging in frequent movements or postural adjustments. It is also speculated that a lower number of postural changes after farrowing could be due to hormonal changes. The release of oxytocin, a hormone that occurs after farrowing, can decrease postural changes in sows. Oxytocin induces relaxation and fosters bonding, potentially resulting in a calmer posture in sows. However, the effect of diet on postural changes remains unclear, warranting further study to assess the relationship between diet and postural changes.

## 5. Conclusions

Increasing feeding allowance during the late gestation period can positively impact both sow and piglet performance, ultimately improving the sustainability and profitability of the swine industry. It significantly improves piglet birth weight, milk yield, and the sow’s physiological condition at weaning. Sows with a high feeding allowance have a lower number of re-estrus days, and their farrowing behavior is not significantly affected. These results suggest that providing sows with adequate nutrients during late gestation is vital for improving piglet growth and overall sow reproductive performance. The findings of this study provide valuable information for optimizing sow nutrition and improving swine production efficiency.

## Figures and Tables

**Table 1:** Feeding management of the experimental animals during the gestation period.

Experimental	Weeks	Days	Feed Amount (kg)
Control (Low Diet)	1–12	1–83	2.50
	12–16	84–114	2.50
Treatment (High Diet)	1–12	1–83	2.50
	12–16	84–114	3.50

**Table 2:** Feeding guide of the experimental sow during the lactation period.

Item	6–1 Days before Farrowing	1 Day after Farrowing	2–6 Days after Farrowing	7–13 Days after Farrowing	14–15 Days after Farrowing	16–28 Days after Farrowing
Feeding amount	2.50/3.50 kg	2.90 kg	3.70 kg	6.00 kg	8.50 kg	9.00 kg

**Table 3:** Physiological conditions of a sows with a low and high feeding allowance during the late pregnancy period.

Parameters	Low Diet		High Diet		SEM	*p*-Value ^#^		
	1st Parity (*n* = 7)	>1 Parity (*n* = 7)	1st Parity (*n* = 7)	>1 Parity (*n* = 7)		D	P	I
**Backfat (P_2_) Thickness (mm) at 84 gestation days**								
Digital	17.29	17.00	17.78	17.43	0.646	0.479	0.702	0.216
Vernier Caliper	17.29	17.14	17.86	17.43	0.586	0.470	0.470	0.062
Body Condition Score	3.07	3.00	2.93	3.07	0.583	0.569	0.569	0.096
**Backfat (P_2_) Thickness (mm) at 107 gestation days**								
Digital	17.36	18.00	18.79	17.64	0.573	0.359	0.666	0.132
Vernier Caliper	17.29	18.00	18.71	17.43	0.570	0.459	0.621	0.092
Body Condition Score	3.07	3.21	3.43	3.18	0.093	0.096	0.569	0.045
**Backfat (P_2_) Thickness (mm) at weaning, 28 days**								
Digital	14.64 ^b^	14.43 ^b^	16.95 ^a^	15.27 ^a^	0.539	0.007	0.091	0.186
Vernier Caliper	14.86 ^b^	14.86 ^b^	16.86 ^a^	15.29 ^a^	0.577	0.046	0.186	0.186
Body Condition Score	2.75 ^b^	2.71 ^b^	3.11 ^a^	2.89 ^a^	0.078	0.002	0.122	0.264
**Backfat Thickness (P_2_) Loss (mm)**								
Digital	2.71 ^b^	3.57 ^b^	1.84 ^a^	2.37 ^a^	0.427	0.022	0.114	0.712
Vernier Caliper	2.43 ^b^	3.14 ^b^	1.85 ^a^	2.14 ^a^	0.270	0.008	0.077	0.436
Body Condition Score	0.32	0.50	0.32	0.29	0.094	0.264	0.456	0.264

^#^ D = diet of sows fed with 2.50 kg (Low) and 3.50 kg (High) from 84 days of gestation to farrowing; P = parity number of sows (1st, primiparous sow; >1, multiparous sow with an average parity number of 3.69); I = interaction (diet × parity) between main effects; SEM = standard error of the mean, ^ab^ letters with different superscripts within a row indicate significant difference.

**Table 4:** Reproductive performances and milk yields of sows with low and high feeding allowances during the late pregnancy period.

Parameters	Low Diet		High Diet		SEM	*p*-Value ^#^		
	1st Parity (*n* = 7)	>1 Parity (*n* = 7)	1st Parity (*n* = 7)	>1 Parity (*n* = 7)		D	P	I
**Sows’ Production Performance**								
Initial weight ^1^	195.29 ^z^	215.71 ^y^	195.00 ^z^	214.29 ^y^	5.313	0.873	0.001	0.915
Final weight ^2^	175.86 ^z^	198.14 ^y^	178.29 ^z^	202.14 ^y^	5.626	0.573	0.000	0.890
Body weight loss (kg)	19.43	17.57	16.71	12.14	2.452	0.110	0.202	0.585
Feed intake (kg)	268.17 ^b^	272.03 ^b^	297.53 ^a^	302.67 ^a^	4.671	0.000	0.744	0.744
Total born (head)	12.86	11.57	13.43	13.29	1.101	0.310	0.523	0.609
Total live birth (head)	11.57	11.00	13.00	12.57	0.898	0.108	0.583	0.937
Mummified piglet (head)	0.29	0.29	0.14	0.29	0.206	0.732	0.732	0.732
Stillbirth (head)	1.00	0.29	0.29	0.43	0.538	0.600	0.600	0.433
Mortality (Piglet; head)	1.00	1.14	1.00	1.00	0.297	0.812	0.812	0.812
Livability, farrowing (%)	93.33	95.92	96.70	94.90	3.801	0.760	0.919	0.569
Livability, weaning (%)	91.26	90.05	92.57	92.24	2.371	0.467	0.750	0.853
Litter size weaned (head)	10.57	9.86	12.00	11.57	0.803	0.062	0.483	0.860
Re-estrus day	6.14	5.57	5.43	5.00	0.311	0.050	0.121	0.820
**Milk Yield (kg)**								
At day 3	14.51	10.27	17.41	15.80	1.510	0.060	0.183	0.544
From days 1–28	294.18 ^b^	296.70 ^b^	362.60 ^a^	360.26 ^a^	0.932	0.014	0.997	0.923

^#^ D = diet of sows fed with 2.50 kg (Low) and 3.5 kg (High) from 84 days of gestation to farrowing; P = parity number of sows (1st, primiparous sow; >1, multiparous sow with an average parity number of 3.69); I = interaction (diet × parity) between main effects; ^1^ Sows were individually weighed at 107 days of gestation days; ^2^ Sows were individually weighed at weaning (28 days); SEM = standard error of the mean; ^ab, yz^ letters with different superscripts within a row indicate significant difference; ^ab^ represent the effects of diet; ^yz^ represent the effects of the sows parity number.

**Table 5:** Average farrowing and distribution intervals of the sows.

Parameters	Low Diet		High Diet		SEM	*p*-Value ^#^		
	1st Parity (*n* = 7)	>1 Parity (*n* = 7)	1st Parity (*n* = 7)	>1 Parity (*n* = 7)		D	P	I
**Farrowing Interval (minutes)**								
Average Farrowing Time	141.71	182.29	168.57	185.86	22.620	0.508	0.213	0.612
Average Farrowing Interval	18.14	20.29	20.14	19.29	3.200	0.877	0.843	0.644
Maximum Farrowing Interval	42.29	65.29	58.71	56.00	14.330	0.805	0.486	0.379
Minimum Farrowing Interval	4.43 ^b^	4.86 ^b^	2.71 ^a^	1.43 ^a^	0.763	0.003	0.579	0.272
**Distribution of Farrowing Interval (head)**								
<10 min	5.71 ^b^	4.71 ^b^	7.43 ^a^	6.86 ^a^	0.871	0.037	0.376	0.808
10–30 min	3.71	3.86	4.14	4.14	0.764	0.644	0.926	0.926
30–60 min	1.57	1.43	0.57	1.29	0.440	0.207	0.523	0.340
>60 min	0.43	0.43	1.00	0.29	0.277	0.446	0.209	0.209

^#^ D = diet of sows fed with 2.50 kg (Low) and 3.50 kg (High) from 84 days of gestation to farrowing; P = parity number of sows (1st, primiparous sow; >1, multiparous sow with an average parity number of 3.69); I = interaction (diet × parity) between main effects; SEM = standard error of the mean; ^ab^ letters with different superscripts within a row indicate significant difference.

**Table 6:** The average frequency and duration of standing in sows with low and high feeding allowances during the late gestation period.

Parameters	Low Diet		High Diet		SEM	Significance of Contrast ^#^		
	1st Parity (*n* = 7)	>1 Parity (*n* = 7)	1st Parity (*n* = 7)	>1 Parity (*n* = 7)		D	P	I
**Frequency of standing**								
Before farrowing ^1^	8.86	11.29	16.43	9.00	1.363	0.064	0.079	0.001
After farrowing ^2^	205.14	212.14	158.71	211.29	3.023	0.214	0.815	0.169
**Duration of Standing (minutes)**								
Before farrowing ^3^	13.14	18.14	13.57	10.00	39.285	0.553	0.456	0.567
After farrowing ^4^	209.86 ^b^	205.86 ^b^	118.43 ^a^	151.43 ^a^	26.958	0.012	0.596	0.499

^#^ D = diet of sows fed with 2.50 kg (Low) and 3.50 kg (High) from 84 days of gestation to farrowing; P = parity number of sows (1st, primiparous sow; >1, multiparous sow with an average parity number of 3.69); I = interaction (diet × parity) between main effects; SEM = standard error of the mean; ^1,3^ collected from the average data per day from 4 days before farrowing until 2 days before farrowing; ^2,4^ collected from the average data per day from 2 days after farrowing until 4 days after farrowing; ^ab^ letters with different superscripts within a row indicate significant difference.

**Table 7:** Interaction of diet and parity in increasing feed allowance during the late gestation period in sows.

Parameter	Diet ^1^	Parity ^2^		SEM	*p*-Value
		1st Parity (*n* = 7)	>1 Parity (*n* = 7)		
**Backfat Thickness (P_2_), 107 days of gestation (BCS)**					
	Low	3.07	3.21	0.931	0.045
	High	3.43	3.18	0.931	
**Frequency of standing before farrowing ^3^**					
	Low	8.86	11.29	1.360	0.001
	High	16.43	9.00	1.360	
**Frequency of standing during farrowing**					
	Low	8.50	8.64	1.321	0.013
	High	16.96	10.07	1.321	

^1^ D = diet of sows fed with 2.50 kg (Low) and 3.50 kg (High) from 84 days of gestation to farrowing; ^2^ P = parity number of sows (1st, primiparous sow; >1, multiparous sow with an average parity number of 3.69); ^3^ frequency of standing one day before farrowing; SEM = standard error of the mean.

**Table 8:** The average production performance of piglets during the lactation phase.

Parameters	Low Diet		High Diet		SEM	Significance of Contrast ^#^		
	1st Parity (*n* = 7)	>1 Parity (*n* = 7)	1st Parity (*n* = 7)	>1 Parity (*n* = 7)		D	P	I
Birth weight (kg)	1.38 ^b^	1.45 ^b^	1.50 ^a^	1.57 ^a^	0.048	0.023	0.163	0.971
Body weight at 3 days	1.67 ^b^	1.68 ^b^	1.81 ^a^	1.87 ^a^	0.064	0.016	0.627	0.719
BWG after 3 days (kg) *	0.29	0.23	0.31	0.30	0.030	0.114	0.230	0.416
Weaning weight (kg)	8.05	8.71	8.63	9.01	0.241	0.079	0.043	0.571
Feed intake/head (kg)	0.40 ^a^	0.40 ^a^	0.34 ^b^	0.32 ^b^	0.008	0.000	0.173	0.644
Body weight gain (kg)	6.67	7.25	7.13	7.44	0.222	0.154	0.055	0.544
Average daily gain	0.24	0.26	0.25	0.27	0.008	0.154	0.055	0.544
Feed conversion ratio	0.059 ^bz^	0.055 ^by^	0.047 ^az^	0.043 ^ay^	0.002	0.000	0.042	0.755

^#^ D = diet of sows fed with 2.50 kg (Low) and 3.50 kg (High) from 84 days of gestation to farrowing; P = parity number of sows (1st, primiparous sow; >1, multiparous sow with an average parity number of 3.69); I = interaction (diet × parity) between main effects; SEM = standard error of the mean; * BWG= body weight gain; ^ab, yz^ letters with different superscripts within a row indicate significant difference; ^ab^ represent the effects of diet; ^yz^ represent the effects of the sows parity number.

## Data Availability

Data presented in this study are available upon request from the corresponding author.
